# On the Religious Instruction of the Insane

**Published:** 1854-01-01

**Authors:** 


### Art. IX.—ON THE RELIGIOUS INSTRUCTION OF THE INSANE.

It is only in Lunatic Hospitals that the course of treatment indicated by an intelligent consideration of the different phases of insanity, can be applied. Nowhere else can the varied forms of occupation, recreation, and amusement be so successfully carried out; nowhere else can the same amount of indulgence be safely granted. It is here alone that beneficial results can be reasonably expected. Elsewhere the consequences will vary with the character of the cases. Generally one of two results ensues—the mind, left to itself, dwells with increasing intensity upon the diseased train of thought, until excitement, growing by its own indulgence, overcomes the vestiges of reason, and the sufferer becomes an ungovernable maniac; or weakened by the shock which disease has inflicted upon the system, gradually loses its power, and sinks into a state of torpidity or childishness. This tendency to mania or dementia, the aggravated extremes of disease, we find illustrated whenever the insane poor of any community are not provided for in a suitable hospital, but are left to the tender mercies of jails and alms-houses, of cages and dungeons, of bonds and fetters. The expositions which have been lately made, of the suffering condition of the pauper insane, in the jails and alms-houses of Massachusetts, and the County houses of New York, when considered in connexion with the energetic philanthropy, and the diligent benevolence of those States, may lead us to fear that a similar investigation would disclose an equally deplorable state of things in Connecticut. Developments occasionally reach us, which would justify the strongest apprehensions of such results.

The religious services of the Sabbath, and of daily evening prayer, have been regularly continued by our estimable chaplain, Rev. Mr. Gallaudet. More extended experience and observation have confirmed the sentiment which I have elsewhere advanced, in relation to the beneficial influence of religious worship; that, leaving out of the estimate all other results, a high rank must be assigned to it as a remedial measure. But its influence may not be thus limited. Amid the vestiges of reason, the affections and the sensibilities exist as warmly and as acute as ever, and in many cases the same high and ennobling results may be attained, as from the operation of similar causes upon individuals under ordinary circumstances. Dr. Poole, the intelligent superintendent of the Montrose Asylum, says, "After the obliteration of reason, many of the highest feelings of our nature remain, to which a successful appeal may be made: and those by which we are connected with a higher sphere of existence admit as readily of being awakened on the proper object being presented to them, as the ordinary passions under which the lunatic acts. Their influence is in the highest degree consoling, and congenial to the return of mental strength and serenity; the effects in each individual are probably as different as in the members of an ordinary congregation." The judicious application of these means, (upon which their efficiency peculiarly depends), requires that they be made, as in the Retreat, in consistency with the general course of discipline and treatment which the medical officer has been led to adopt. Having for a long time been deeply impressed with a sense of the duty and importance of providing for the religious instruction of the insane, it is gratifying to witness the extending prevalence of similar sentiments. The claims of the lunatic, that he shall no longer be excluded from the privilege of worshipping God, are being widely recognised. In all the leading institutions of this country, and in the Retreat among the first, arrangements have been made for the due and customary observance of the Sabbath, either by the appointment of a chaplain, or by the aid of the neighbouring clergymen. In most, suitable chapel accommodations are provided, to add force and dignity to the service. The same is true of many of the hospitals in Great Britain and on the Continent.

Thus, one by one, are the errors and delusions relative to insanity passing away; the enlightened reason and the sympathy of their fellow-men are opening the prison-doors of the lunatic, striking off his fetters, and restoring the rights and dignity of humanity. They are giving back to him the Sabbath day, that all the calm and soothing influences of that holy time may rest upon his heart.—From Dr. Butler's interesting *Report of the Hartford Lunatic Asylum, U. S. America.*

